# Parents Value Demonstration as a Determinant of Youth Experiences and Responses to Parents’ Warnings Following the Onset of Risk Behavior

**DOI:** 10.1007/s10964-025-02196-7

**Published:** 2025-07-16

**Authors:** Avi Assor, Rinat Cohen, Wendy Grolnick, Judith G. Smetana, Efrat Sher-Censor, Noam Itshaki

**Affiliations:** 1 Educational Psychology program, School of Education, Ben-Gurion University, Beersheba, Israel; 2 School of Education, Achva Academic College, Yinon, Israel; 3 Department of Psychology, Clark University, Worcester, MA USA; 4 Department of Psychology, University of Rochester, Rochester, NY USA; 5 The School of Psychological Sciences and the Center for the Study of Child Development, The University of Haifa, Haifa, Israel

**Keywords:** Parenting adolescents, Problem behavior, Inherent value demonstration, Perspective taking, Need frustration/support, Warnings

## Abstract

When parents first learn about their adolescent’s problem behaviors, they may warn their teen that further involvement in problem behaviors will lead to increased restraints, surveillance, or resource withdrawal. However, research has not investigated how adolescents experience and respond to such warnings. Drawing on research on the benefits of parents’ demonstration of the merit of their values in their behavior (inherent value demonstration), this study examined the potential role of inherent value demonstration as a moderator of youth responses to warnings. Participants were 105 Israeli adolescents (*M*age = 14.87 years, *SD* = 1.52, 57.1% female) who completed an individualized survey asking them to indicate which of 29 problem behaviors they had engaged in during the last month. The survey then selected the most serious problem behavior youth engaged in, and asked them to rate two parental reactions - warnings and perspective-taking - to the onset of this behavior, their experiences and responses following parents’ reactions, problem behavior recurrence, and parents’ general inclination to demonstrate their values in their behavior. As hypothesized, and with youth problem behavior characteristics and parents’ perspective-taking controlled, youth were more likely to experience their parents’ warnings as need-frustrating and respond defiantly when they perceived their parents as failing to demonstrate their values in their behavior. Additionally, inherent value demonstration was positively associated with perception of parents’ reactions as need supporting and negatively related to perception of parents reactions as need thwarting and defiance. These findings suggest that parents’ inherent value demonstration may function as a protective factor that enables youth to experience their parents’ warnings less negatively.

## Introduction

As part of the transition to adolescence, many youth start to experiment with problem behaviors such as alcohol and drug use, school truancy, vandalism, and other rule-breaking behaviors (Lacourse et al., 2003; Willoughby et al., 2021). A common parental reaction to finding out about youth involvement in problem behavior is warning youth that if they continue to engage in the problem behavior, the parents will increase their surveillance, remove resources, and apply more rules (Sher-Censor et al., 2021). Presently, it is not clear how youth experience and respond to such warnings. Thus, youth may experience the warnings as controlling coercive acts and respond in a defiant manner, or alternatively, they may experience warnings as non-controlling attempts to protect them from trouble. Grounded in self-determination theory (Ryan & Deci, 2017), this study examined the role of parental inherent value demonstration (Assor et al., 2023) as a general parental attribute that may affect whether youth experience warnings following misbehavior as controlling parenting, which undermines their basic needs and evokes a defiant response.

### Parenting Practices Associated with Youth Problem Behaviors

Research shows that a number of parental practices can serve as protective factors for various problem behaviors, especially when they occur together. For example, researchers have examined the role of authoritative parenting (e.g., Baumrind, 2005), parental knowledge of adolescents’ activities (e.g., Kapetanovic et al., 2019; Kerr et al., 2010), parental warmth and involvement (Fletcher et al., 2004; Pinquart, 2017), parents’ support at school (Walsh et al., 2010), behavioral control, including clear expectations and consistent consequences for behavior (Soenens et al., 2006), which Grolnick et al. (2014) have viewed as parental structure, inherent value demonstration (Assor et al 2020b; Yu et al., 2023), applying logical constraints (Robichaud et al., 2020) and parents’ thwarting of adolescents’ basic psychological needs (Soenens et al., 2019). These aspects of parenting are all considered important in preventing problem behavior and have been well-studied.

There is very little research, however, on what parents ***do*** once problem behavior has occurred. That is, how parents react when they *first learn* about their adolescents’ involvement in problem behaviors, and in turn, how adolescents experience these parental actions and respond to them. Youth experiences and responses following parents’ initial reactions may be important because they may set the direction of further adolescent-parent dynamics. For example, youth may experience their parents’ reactions to the onset of problem behavior as frustrating their basic psychological needs, leading to defiance and further engagement in problem-behavior.

Parents may take a number of actions after finding out for the first time that their youth has engaged in problem behavior. For example, they may try to take the adolescent’s perspective and have a conversation to try to understand and address the reasons for this behavior (Lundell et al., 2008; Marbell-Pierre et al., 2019; Soenens et al., 2019).‏ Parents may also try to influence their adolescents via surveillance or by increasing the stringency of rules about where children can go and what they can do, or by taking away resources, such as access to money or cell phones. Alternatively, parents may refrain from initiating such responses, and “only” warn children that if they continue the behaviors they will take these more controlling actions.

Considerable research shows that coercive and highly controlling parental behavior contributes to adolescents’ engagement in problem behaviors and defiance towards parents (e.g., Rodríguez-Meirinhos et al., 2020; Soenens et al, 2009; Vansteenkiste et al., 2014). In contrast, parental perspective-taking is likely to be negatively associated with problem behaviors (Psychogiou et al., 2008), especially when combined with other positive parental behaviors, such as provision of choice or lack of coercive controlling parenting (Soenens et al., 2007), or clear expectations (e.g., Sher-Censor et al., 2015). However, there is little research on parental warnings, although this practice may appear natural to many parents and is quite common (e.g., Sher-Censor et al., 2021). Thus, more research is needed on how adolescents experience and respond to parental warnings.

### The Possible Effects of Parents’ Warnings Following the Onset of Youth Problem Behavior

An understanding of how adolescents experience and respond to parental warnings following the onset of problem behaviors can be guided by different theoretical perspectives such as Jessor’s (2018) problem behavior theory, or more general theories of parenting (e.g., Steinberg et al., 2013). These well-studied perspectives have focused primarily on the parenting factors that help reduce the incidence of problem behavior, but they do not offer much guidance as to how youth may experience their parents’ warning reactions after they have discovered youth engagement in problem behavior.

The present study examined this issue through the framework of self-determination theory (Ryan & Deci, 2017), which includes an elaborate conceptualization of controlling parental behaviors and youth experience and response to such behaviors. Self-determination theory posits that individuals have innate needs for autonomy (the need to feel free to realize their authentic preferences rather than feel coerced and pressured), competence (the need to feel able to cope with challenges and avoid undesirable consequences), and relatedness (the need to feel closely connected with people one cares about). Studies have shown that coercive and controlling parental practices are experienced by youth as need-thwarting and potentially evoke defiance (van Petegem et al., 2017). Thus, parents may view warnings that they will institute more surveillance, rules, and resource removal as a logical, relatively benign first step to prevent further engagement in problem-behavior. However, adolescents may vary in their responses to these warnings. Some may experience parental warnings as a coercive practice that thwarts their basic psychological needs, thereby evoking defiance (Ryan & Deci, 2017). Others, however, may experience warnings as an aspect of need-supportive parental structure involving clear expectations, guidelines, and consequences for action (e.g., Grolnick & Pomerantz, 2009). Structure supports people’s need for competence because it helps them attain their goals and avoid negative consequences. Therefore, it is possible that youth will interpret warnings, such as that they will have to return home early if they keep going to unsafe places, as a parental reaction that helps them avoid harmful contexts and consequences.

Furthermore, social-cognitive domain theory suggests that adolescents’ reactions to their parents’ interventions may depend on the type of transgression committed (Smetana, 2018; Turiel, 1983). Parents and adolescents differentiate among immoral behaviors (e.g., those that are unfair, unjust, or cause harm to others), behaviors that cause prudential harm (e.g., those that are dangerous or unsafe and cause harm to oneself), conventional issues (i.e., contextually relative norms that facilitate smooth social functioning; for example, customs for greeting others, such as bowing or shaking hands), and personal behaviors that involve control over one’s body, privacy, and personal preferences and choices. Parents and adolescents believe that it is more legitimate for parents to intervene when adolescents commit moral or prudential offenses than when they engage in conventional misbehavior or personal choices (Smetana, 2011, 2018). Problem behaviors often involve moral and/or prudential harm. Therefore, youth may view warnings following problem behavior as legitimate and reasonable, particularly if the problem behavior involves harm to others or the self. Consequently, such warnings may be experienced as less need thwarting than when they commit other types of transgressions and are therefore less likely to lead to defiance.

This reasoning is supported by a study examining the effects of parents implementing structure in autonomy supportive or controlling ways across different spheres: academics, home responsibilities, and unsupervised time (Grolnick et al., 2015). When structure was implemented in a controlling manner regarding academics and responsibilities, it undermined children’s autonomous regulation of behavior, perceptions of competence, and engagement in trying to follow the rules. However, this was not the case for unsupervised time, perhaps because the latter case involved the possibility of harm (e.g., adolescents being bullied or getting lost). Therefore, youth may not have perceived the structure as being controlling and instead may have viewed it as legitimate and rightfully up to parents to step in without first asking for youth’s opinions. By contrast, as they saw academics and home responsibilities as falling within the personal or conventional domains, they may have experienced the structure as more controlling and responded negatively.

Only one previous study has examined the effects of parents’ warnings after finding out that their adolescent had begun to engage in problem behavior (Sher-Censor et al., 2021). This study showed that when parents found out that their children hung out with peers who had engaged in serious immoral and prudentially problematic behaviors, their warnings were followed by reduced association with these peers, but not with more disclosure and consultation regarding problem behaviors. Similar to the reasoning just discussed, adolescents may view warnings in such situations as a legitimate type of intervention (Sher-Censor et al., 2021).

While these findings are interesting, the immoral or non-prudential problem behaviors studied were extreme (e.g., hanging out with peers who hurt animals, engaging in excessive drug use) and were described to the participants as dangerous, immoral, or illegal. Describing peers’ problem behaviors in this way underscored their immoral and harmful nature and therefore might have made parental warnings more acceptable. However, warnings might be less acceptable when they are applied to mild or less extreme problem behaviors that are more common (e.g., skipping or missing classes without permission, hanging out past curfew, behaving rudely toward older people, or lying to parents).

### The Role of Inherent Value Demonstration in Youth Experiences of and Responses to Parental Warnings

As suggested above, adolescents may experience parents’ warnings after learning that their adolescent engages in problem behaviors in very different ways. While for some, warnings might be seen as need thwarting, for others, they may not. One factor that may affect these perceptions is the youth’s more general view of their parents. The present study focused on one perceived parental attribute: inherent value demonstration (Cohen et al., 2024). This concept refers to youth perception of their parents as behaving in ways that are consistent with their endorsed principles and as feeling vital, focused, and standing fully behind their principles as they enact them. For example, parents who talk to their children about the importance of helping others in need demonstrate a high level of inherent value demonstration if they participate in various helping activities, and while doing this appear to be fully committed and serious about these activities. Youth perceived parental inherent value demonstration was found to be associated with a number of positive socialization outcomes, including autonomous internalization of parental values (Brambila et al., 2015), resistance to negative peer pressure (Assor et al., 2020b), avoidance of cheating (Yu et al., 2023), and low levels of depressive feelings (Cohen et al., 2024).

When parents consistently demonstrate their stated values in their ongoing behavior, children are more likely to respect them and perceive them as sincere and trustworthy. As a result, they may also be more inclined to view parents’ warnings as non-controlling trustworthy attempts to protect them, even when these attempts have some unpleasant aspects (e.g., resource removal). Consequently, warnings initiated by parents showing high levels of inherent value demonstration may not be experienced as need thwarting. In contrast, when adolescents perceive their parents as lacking a clear, authentic and consistent value orientation (i.e., as low on inherent value demonstration), they are less likely to attribute their parents’ warning reaction to their solid value orientation. Instead, they may attribute parents’ warnings to their anger or lack of faith in the adolescent’s capacity or intention to stay away from trouble. As a result, the warnings of parents exhibiting low inherent value demonstration are more likely to be experienced as need-thwarting and to evoke defiance. Thus far, however, the role of inherent value demonstration in youth experiences of and responses to parental warnings has not been examined.

**Fig. 1:**
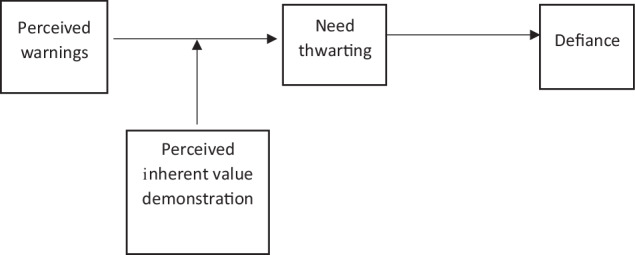
Inherent value demonstration as a moderator of the mediation path leading from warnings to defiance through need thwarting

The foregoing considerations suggest that inherent value demonstration may serve as a moderator of the link between parental warnings following the onset of youth problem behaviors and youth experience of parents’ reactions as need thwarting. Furthermore, because need-thwarting parenting often leads to defiance (Vansteenkiste & Ryan, 2013; Van Petegem et al., 2017), the experience of need thwarting following parental warnings is likely to be followed by a defiant response toward parents. Accordingly, it can be posited that inherent value demonstration would moderate the mediation path from warnings to defiance through need thwarting. These associations are depicted in Fig. 1. The main aim of our study was to test this conceptual model.

In addition to its role as moderator of the link between warnings and defiance through need thwarting, parental inherent value demonstration may also be associated directly with positive youth experiences and responses. Specifically, perceived parental inherent value demonstration may be associated with perceptions of parents’ reactions as need-supporting and with youth’s attempts to cease problem behavior. Youth whose parents behave consistently according to their stated values are likely to respect and have a more positive perception of their parents and in turn, may be inclined to view their parents’ reactions as positive need-supporting acts. In addition, they may also be more likely to cease behaviors which may worry the parents they respect.

### Parental Perspective Taking and Problem Behavior Recurrence

Many parents may react to their teenager’s first (known) engagement in problem behavior with warnings. However, others may try to take their youth’s perspective in a way that respects their reasoning and feelings (e.g., Ryan & Deci, 2017). Parents who react with perspective-taking may do so because they want to understand why their youth started to engage in the problem behavior before they initiate any further response. Therefore, they may not issue warnings. In this case, low scores on warnings may simply reflect parents’ perspective-taking as an alternate strategy from issuing warnings. Additionally, parents’ empathic perspective-taking may reduce the negative effects of warnings because this may cause the child to feel that the warnings are not an attempt to control them, but rather are motivated by genuine interest and concern for the child. Therefore, main and interactive effects of youth perception of parents’ perspective-taking reaction to the onset of the problem behavior were controlled in the analyses.

Youth’s experiences and responses to parental warnings may also be influenced by the extent to which the target problem behavior (i.e., the behavior triggering the warnings) is a recurrent misbehavior. When these are recurring transgressions that started months ago, the pattern of enduring and repeated transgressions may partly reflect parents’ lack of success in influencing youth’s problem behaviors (e.g., Guilamo‐Ramos et al., 2006; Ream & Savin-Williams, 2005) and hence may reflect a previously established defiant response to parents. Thus, when recurrence is high, parents’ warnings may affect youth less, because they pay less attention to what parents say or do. Therefore, in testing the hypothesized model, the possible effects of problem behavior recurrence were also controlled for.

## The Current Study

Thus far, research has not examined the role of parents’ inherent value demonstration as a moderator of youth experience of and response to parents’ warnings following the onset of youth misbehavior. Given this research gap, the main objective of the present study was to test a conceptual model of the role of parents’ inherent value demonstration as a determinant of youth experience and response to parents’ warnings, as shown in Fig. 1.

According to this model, youth-perceived parental inherent value demonstration was expected to moderate the link between youth-perceived parental warnings in reaction to the onset of problem behavior and youth experience of need thwarting, and in turn, defiant responses to parents. Specifically, it was expected that the link between warnings, need thwarting, and subsequent defiance would be stronger for youth perceiving their parents as lower on inherent value demonstration. Furthermore, it was expected that these associations would hold even when controlling for the effects of parent’s perspective taking reaction and for the effects of problem behavior recurrence. A secondary objective of this study was to examine whether youth-perceived parental inherent value demonstration is associated positively with experiencing parents’ reactions as need satisfying and with youth’s attempts to cease problem behavior.

## Method

The study procedure and measures were approved by the Human Research Review Board of the participating university and the chief scientist of the Ministry of Education. The procedures used in this study adhere to the tenets of the Helsinki declaration.

### Procedure

Participants were given a list of 29 problem behaviors (presented in Table 1 of the supplementary materials). Most of the items were based on scales used by Elliott et al., (1985), and modified versions of these scales (Cho et al., 2010; Loeber et al., 1993), and some items were adapted from Kakihara et al., (2010) rule breaking scale. For each, they indicated whether they engaged in it in the last month and whether their parents knew about it. Then, based on the severity index established in a pilot study (presented in the supplementary materials), a specially designed computer program selected the most severe behavior the participant reported that their parents found out about in the last month. The rest of the questionnaire focused only on that behavior, which in in this paper is termed *youth’ individualized problem behavior*. In the next phase, participants reported whether they had engaged in that behavior once or more, when the first time this behavior was performed, and when the parents first became aware of their involvement in this behavior (i.e., recurrence information). Next, adolescents rated the extent to which their parents enacted warnings and perspective-taking reactions to their individualized problem behavior. This was followed by questions assessing the extent to which participants attempted to cease the individualized problem behavior, whether they engaged in defiant behavior in response to their parents’ reactions, and the degree of need support or thwarting they felt following their parents’ reactions. Finally, adolescents reported on their parents’ general inherent value demonstration and on socio-demographic variables.

### Participants

The initial sample included 182 7^th^ to 10^th^ grade Jewish adolescents (*M*age = 14.66, *SD* = 1.54, range = 12 – 17, 56% female). Of this sample, 105 participants (*M*age = 14.87, *SD* = 1.52, range 12 – 17, 57.1% female) constituted the final sample, as they reported engaging in at least one problem behavior in the last month. The participants were recruited from WhatsApp groups of students from different classrooms and grade levels in three small towns in southern Israel. Participants were offered a modest monetary compensation for participating in the study and were assured that the data were highly confidential. The participants who did not engage in any problem behavior did not differ from the final sample in terms of gender (χ^2^ = 0.75, *p* = 0.68), age (*t* = 1.52, *p* = 0.13) and mothers’ (*t* = 0.36, *p* = 0.72) and fathers’ (*t* = 0.63, *p* = 0.82) education.

To assess statistical power, a power analysis was conducted using G*Power 3.1 (Faul et al., 2009) to calculate the required sample size for multiple regression analysis with seven predictors (warnings, perspective-taking, the interaction between inherent value demonstration and warnings, the interaction between inherent value demonstration and perspective taking, problem behavior recurrence, and parental education). This analysis also applies to the moderated mediation analysis which included fewer variables. Following conventions in social science research (Cohen, 1988), a medium effect size of f^2^ = 0.15, an alpha level of 0.05, and a desired power of 0.80, were assumed. The results indicated that a minimum sample size of N = 103 is required to achieve the power coefficients noted above. Given the current study’s sample size of 103, the main analysis achieved a power of 0.81, demonstrating that the sample size is sufficient to ensure statistical power above the accepted threshold ( > 0.80).

Participants reported on each parent’s educational background on a seven-point scale, ranging from 1 (elementary school) to 7 (doctoral degree). Most of the participants came from families where at least one of the parents had at least a college education (61% of fathers, and 68% of mothers). For the analyses, mothers’ and fathers’ scores were averaged. Parents in most families were college educated.

Because of time limitations it was not possible to assess participants’ perceptions and responses to both parents. As a result, participants were asked to refer to the parent who is more involved in their life. Fifty-six percent of the participants reported that both parents were equally involved and present in their lives, 40% reported that their mother was more involved, and 4% rated their fathers as more involved. The participants answered the questions for both parents if they were described as equally involved and for one parent if that parent was described as more involved. As shown in the results section, and in the supplemental materials, it was found that the identity of the parental figures did not have a significant main effect on the outcome measures, nor did it moderate the effects of any predictor variable (warnings, inherent value demonstration, etc.) on youth experiences and responses (i.e., no significant interaction effects).

### Measures

All items were rated on a five-point Likert-scale (1 = *completely agree*; 5 = *completely disagree*). Scales were translated from English to Hebrew. The section below presents the measures assessing the main variables of the study.

#### Perceived parental reactions

Participants reported on their parents’ responses to the onset of their involvement in their individualized problem behavior using two scales that reflected two types of parental reactions: warnings to use active control and perspective taking. Items were adopted mainly from Sher-Censor et al., 2021). All items are presented in Table 2 in the supplementary material, which also includes the results of a confirmatory factor analysis (CFA) supporting the construct validity of these scales. Four items measured perceived ***parental perspective taking*** (e.g., “My parents really tried to understand what I was feeling and what made me act this way”), α = 0.77. Four items measured parental use of ***warnings to apply to apply more surveillance, rules and resource-removal*** (e.g., “My parents said that if I continue to behave this way, they will have to ask me to give them detailed information about what I do, who I hang out with, where I go, and when I return”), α = 0.82.

#### Perceived parental inherent value demonstration

Using a previously validated nine-item scale (Assor et al., 2021; Yu et al., 2023), participants rated how much each item reflects their parents’ general behavior (e.g., “My parents not only talk about what is important to them but also show it in their behavior,” “When my parents act in ways that fit their values—they look satisfied and full of energy”). Scale alpha was 0.86.

#### Adolescents’ experiences of parents’ reactions as need-supporting or thwarting

Adolescents’ experiences of need support and thwarting following parents’ reactions to their most serious recent individualized problem behavior were measured using 14 items based on the Basic Psychological Need Satisfaction and Frustration Scale (BPNSFS; Chen et al., 2015) and on Sher-Censor et al. (2021). All items began with the stem, “What did you feel and think after the last time your parents reacted as you described in the previous section?” For each item, participants were presented with their individualized problem behavior (their most severe problem behavior) and then were asked to indicate their need-related experience of their parents’ reactions to this behavior. ***Need thwarting*** was assessed by seven items (three autonomy, two relatedness, and two competence, *α* = 0.76). Item example: “I felt that I am being forced to do things I would not really choose to do.” ***Need support*** was assessed by 7 items (three autonomy, two relatedness, and two competence; *α* = 0.84), e.g., “I felt that I can decide and act in a way that reflects what I truly want.”

#### Adolescents ceasing the individualized problem behavior

This variable was measured on a 10-item scale based on Sher-Censor et al. (2021). Items began with the stem, “What did you do, think, and feel after the last time your parents reacted as you described in the previous section?” For each item, participants were presented with their individualized problem behavior and then rated their agreement that following the last time their parents reacted in the ways they described in the previous section, they ceased this problem behavior (5 items) or continued to do it (5 reversed score items), e.g., “I stopped behaving in this way.” Alpha was 0.84.

#### Adolescents’ defiant response to parents

Defiance was measured via a three-item scale (Sher-Censor et al., 2021). Items began with the stem, “What did you do, think, and feel after the last time your parents reacted as you described in the previous section?” For each item, participants rated whether, following the last time their parents reacted in the ways they described in the previous section, they wanted to act in the way the item described (e.g., “I wanted to do the exact opposite of what my parents wanted me to do.”). Alpha was 0.84.

#### Problem behavior recurrence

To assess the recurrence of the individualized problem behavior, participants indicated when they started to engage in the problem behavior, and how many times they engaged in this behavior in the last six months. Based on the response distributions, a recurrence indicator was created, consisting of three levels: Low recurrence (participants engaged in the behavior for the first time once, during the last week or two), medium recurrence (participants engaged in the behavior 2–3 times in the last month), and high recurrence (participants engaged in the behavior more than three times and started to enact the behavior prior to the last month). As could be expected, recurrence of the individualized problem behavior was positively and significantly correlated with the number of problem behaviors engaged in during the last month. and with the youth age (see Table 1 in the supplementary materials). Thus, participants who repeatedly engaged in problem behaviors were also more inclined to engage in more problem behaviors in the last month.

### Analytic Plan

Preliminary analyses were conducted in two steps. First, the factorial structure of the items assessing perceived parental responses to problem behaviors was examined using confirmatory factor analyses (CFA), based on the data collected in the pilot study. Second, based on the data collected in the main study, means, standard deviations, and inter-correlations were computed for all study variables. In the main analyses, the first objective of the study was addressed by testing the hypothesized model depicted in Fig. 1 via a moderated mediation analysis. Then, the second objective was addressed by examining the expected positive correlates of inherent value demonstration by means of regression analyses. In addition, a supplementary materials section reports regression analyses assessing the robustness of the study findings. Specifically, we examined whether the results obtained in testing the study hypotheses hold when controlling for the effects of problem behavior severity and number of problem behaviors, as well as demographic and method variables that may be related to the main variables of the study. In the supplemental materials we present more detailed analyses.

## Results

### Preliminary Analyses

#### Measurement model (CFA) for the perceived parental variables

We conducted a confirmatory factor analysis (CFA) using AMOS‐25 (Arbuckle, 2017) to ascertain that the items measuring warnings, perspective taking, and inherent value demonstration were perceived as reflecting distinct and coherent constructs. The measurement model of the CFA was composed of these variables, modeled as three latent factors. Except for inherent value demonstration, each latent variable included four items. We parceled the inherent value demonstration items to create a reasonable ratio of observed indicators with respect to the sample size (Bandalos & Finney, 2001). There were three parcels, with three items for each inherent value demonstration parcel selected randomly. The results indicated an adequate fit to the data, χ2(41) = 88.29, *p* < 0.001, CFI = 0.90, RMSEA = 0.08. Items and parcel loadings onto their respective factors were all satisfactory, statistically significant, and ranged from 0.60 to 0.92. The items and their loadings are presented in Table 2 of the supplementary material. These findings indicate that these three variables measured distinct constructs.

#### Descriptive statistics, reliability, and correlations among research variables

Table 1 presents the means, SDs, internal reliabilities, and bivariate correlations among the study variables. As expected, perceived parental warnings had positive and significant correlations with adolescent need thwarting and defiance. Perceived parental perspective-taking had positive, significant correlations with inherent value demonstration, adolescent cessation of problem behavior, and need support, and had significant negative correlations with defiance and need thwarting. Importantly, perspective taking was the only parental variable showing a significant association with ceasing the individualized problem behavior. As expected, inherent value demonstration had a positive and significant correlation with need support and a significant negative correlation with defiance. Also as expected, higher levels of inherent value demonstration were linked to lower levels of need thwarting and defiance, and were positively related to need support, but not to ceasing the individualized problem behavior. Unexpectedly, parents’ education was significantly correlated with less need support and more need thwarting.

**Table 1:** Descriptive statistics, reliability, and correlations among study variables (N = 105)

	Mean	SD	α	1	2	3	4	5	6	7	8	9	10	11	12	13
1. Warnings	1.94	1.11	0.82	1												
2. Perspective taking	3.21	1.23	0.77	−0.30^**^	1											
3. Inherent value demonstration	4.21	0.69	0.86	−0.07	0.28^**^	1										
4. Ceasing problem behavior	2.90	0.92	0.87	0.07	0.23^*^	0.07	1									
5. Need support	3.38	0.99	0.84	−0.14	0.45^**^	0.35^**^	0.26^**^	1								
6. Need thwarting	1.88	0.73	0.76	0.50^**^	−0.24^*^	−0.39^**^	0.04	−0.38^**^	1							
7. Defiance	1.84	1.01	0.84	0.42^**^	−0.26^**^	−0.34^**^	−0.14	−0.34^**^	0.66^**^	1						
8. Recurrence	1.49	0.57		0.07	−0.12	−0.15	−0.18†	−0.03	0.04	0.22*	1					
9. Severity of problem behavior	4.91	0.97	0.87	−0.04	0.05	−0.13	0.16	−0.03	0.02	0.12	0.01	1				
10. Number of risk behaviors	4.78	3.60	0.80	0.21^*^	−0.18^~^	−0.32^**^	−0.06	−0.32^**^	0.36^**^	0.42^**^	0.17^*^	0.35^**^	1			
11. Child age	14.66	1.54		0.18	−0.12	−0.05	−0.20^*^	0.003	0.05	0.25^*^	0.22^*^	−0.03	0.13	1		
12. Child gender ^a^				0.09	−0.16	−0.02	0.08	−0.10	0.11	0.11	0.01	0.001	0.03	0.09	1	
13. Parents’ education	4.85	1.40		0.16	−0.13	−0.07	−0.09	−0.28^**^	0.34^**^	0.15	−0.004	0.008	0.07	0.07	0.04	1

^a^Child gender is coded as follows: 0 = male (58.3%), 1 = female (41.7%)

†*p* < 0.08, *p < 0.05, **p < 0.001

### Main Analyses

#### Tests of the hypothesized conceptual model

First, we tested the hypothesized model (see Fig. 1). The model posited that youth-perceived parental inherent value demonstration moderates the link between parental warning reactions to the onset of problem behavior and youth experience of need thwarting, and subsequent defiant response to parents. We expected that the model would also hold when controlling for the effects of the parenting reaction of perspective taking, problem behavior recurrence, and parents’ education.

A full test of the moderated mediation model (Morgan-Lopez & MacKinnon, 2006) depicted in Fig. 1 requires showing that the link between warnings and defiance is mediated by need-thwarting. Furthermore, the model also requires that inherent value demonstration will moderate the link between parents’ warnings and need thwarting, but not the link between need-thwarting and defiance. To test our moderated mediation model, we used model 59 of Hayes’ (2018) SPSS PROCESS macro program.

**Fig. 2:**
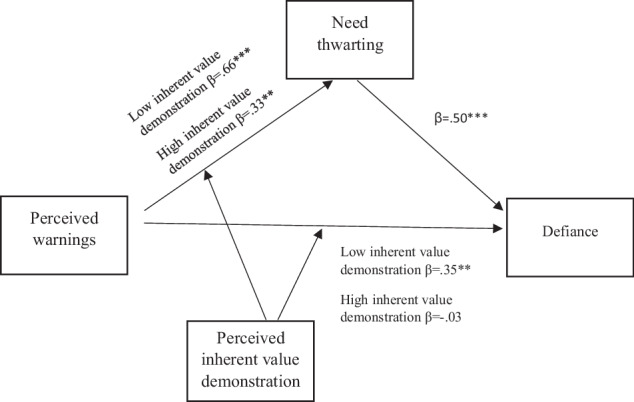
Inherent value demonstration as a moderator of the path from warnings to defiance through need thwarting and of the direct path from warnings to defiance. *Note*: Perspective taking, problem behavior recurrence, need support, and parents’ education were included in the model as covariates. *p < 0.05, **p < 0.001.

Accordingly, we included warnings as the independent variable, need thwarting as the mediating variable, and defiance as the dependent variable. To test the prediction that inherent value demonstration would moderate the link between warnings and need thwarting, but not the link between need thwarting and defiance, we included inherent value demonstration as moderator of both links. In addition, we examined the possibility that inherent value demonstration would moderate the direct link between warnings and defiance. Perspective taking, problem behavior recurrence, need support and parents’ education were included in the model as covariates. The results of the moderated mediation analyses are presented in Table 2 and in Fig. 2.

**Table 2:** Results of the moderated mediation analysis of the path from perceived parents’ warnings to youth defiance through youth experience of need thwarting

	Adolescent need thwarting (mediator)				
	*B*	*S.E*	*t*	*p*	*F*
Warnings	0.33	0.05	6.29	<0.001	*F*(7, 91) = 13.93, *p* < 0.001, *R*^*2*^ = 0.52
Inherent value demonstration	−0.33	0.08	−3.88	<0.001	
Warnings * Inherent value demonstration	−0.15	0.07	−2.14	0.03	
Perspective taking	0.07	0.05	1.28	0.20	
Parents’ education	0.11	0.04	2.51	0.01	
Recurrence	0.01	0.09	0.20	0.83	
Need support	−0.16	0.06	−2.34	0.02	

	Adolescent defiance (outcome)				
	*B*	*S.E*	*t*	*p*	*F*
Warnings	0.15	0.08	1.74	0.08	*F*(9, 89) = 11.98, *p* < 0.001, *R*^*2*^ = 0.55
Need thwarting	0.69	0.14	4.60	<0.001	
Inherent value demonstration	−0.13	0.12	−1.04	0.29	
Warnings * Inherent value demonstration	−0.24	0.11	−2.02	0.04	
Perspective taking	0.01	0.06	0.17	0.86	
Recurrence	0.35	0.12	2.72	0.007	
Need support	−0.12	0.08	−1.31	0.19	
Parents’ education	−0.06	0.05	−1.10	0.27	
Need thwarting * Inherent valuedemonstration	−0.02	0.17	−0.15	0.87

The overall moderated mediation model was supported by a satisfactory index of moderated mediation (β = −0.08, 95% CI [−0.18, −0.01]). The conditional indirect mediation effect was significant for those who perceived parental inherent value demonstration as low (β = 0.34, 95% CI [0.17, 0.61]), average (β = 0.25, 95% CI [0.12, 0.42]), and high (β = 0.16, 95% CI [0.03, 0.31]). As expected, need thwarting functioned as a stronger mediator of the relation between warnings and defiance when youth perceived their parents as low rather than average or high on inherent value demonstration.

Further analyses examined the role of inherent value demonstration in moderating the three links depicted in our conceptual model. First, we examined the conditional effect of warnings on need-thwarting (moderated by inherent value demonstration). This effect was found to be significant (β = -0.16, p = 0.03). Tests of the conditional effects of warnings on need-thwarting at three levels of inherent value demonstration showed that they were all significant: for low inherent value demonstration (β = 0.66, t = 5.79, p < 0.001), for medium inherent value demonstration (β = 0.49, t = 6.29, p < 0.001), and for high inherent value demonstration (β = 0.33, t = 3.15, p = 0.002). Thus, as expected, need thwarting functioned as a stronger mediator of the relations between warnings and need thwarting, the more youth perceived their parents as low on inherent value demonstration. The second conditional effect examined the link between need thwarting and defiance (moderated by inherent value demonstration). As expected, this effect was not significant (β = −01, p = 0.87). As the conditional effects test showed that this effect was not significant, Fig. 2 reports the overall beta for this link (β = 0.50, p < 0.001). Finally, we examined the conditional effect of warnings on defiance (moderated by inherent value demonstration). This effect was significant (β = -0.18, p = 0.04). Tests of the effects of warnings at three levels of inherent value demonstration showed significant associations for low inherent value demonstration (β = 0.35, t = 2.51, p = 0.01) and for medium inherent value demonstration (β = 0.16, t = 1.74, p = 0.05), but not for high inherent value demonstration (β = -0.03, t = 0.18, p = 0.85). The findings pertaining to the direct link between inherent value demonstration and defiance suggest that need thwarting is a partial mediator of the link between warnings and defiance, so that warnings had both indirect and direct links to defiance.

#### Tests of the hypothesized positive correlates of inherent value demonstration

To address the second study objective, we conducted regression analyses testing whether, as expected, inherent value demonstration would be positively associated with youth experience of parents’ reactions as need supporting, and with ceasing the problem behavior. To examine whether the expected positive associations of inherent value demonstration would be observed also when controlling for the parenting reactions of warnings, perspective taking, and their interactions with inherent value demonstration, we included the latter variables in the regression analyses. In addition, we also included problem behavior recurrence and parental education. Parent education was included as a predictor because, as shown in Table 1, it was correlated significantly with youth need experiences following parents’ reactions. To get a fuller picture, we examined not only the expected positive correlates of the experience of need support and ceasing the problem behavior, but also the negative correlates of the experience of need thwarting and defiance.

Accordingly, analyses were performed on each of the four outcome variables: youth experience of parents’ warning reactions as need-thwarting or need supporting, youth defiance of parents, and youth ceasing the problem behavior. Each regression analysis examined the effects of the following predictors: warnings, inherent value demonstration, the interaction between warnings and inherent value demonstration, perspective taking, the interaction between perspective taking and inherent value demonstration, problem behavior recurrence, and parents’ education.

The results of the four regression analyses (one for each outcome variable) are summarized in Table 3. For the full results of each of the regression analyses, see Tables 3–6 in the Supplementary materials. As expected, inherent value demonstration was positively associated with need support, but unexpectedly, it was not associated with ceasing engagement in the problem behavior. Perspective taking was associated with need support and ceasing the problem behavior. However, perspective taking did not interact with warnings to affect youth experiences and responses. Thus, the negative effects of warnings were observed irrespective of the extent to which youth perceived their parents as taking their perspective after finding out about their involvement in problem behavior. Also as expected, problem behavior recurrence was associated with defiance. Parents’ education was significantly associated with less need support and more need thwarting.

**Table 3:** Regression analyses predicting adolescents’ experiences of need support and need thwarting, ceasing the problem behavior, and defiance

Predictors	Need support	Need thwarting	Defiance	Ceasing the problem behavior
Warnings	0.03	0.49***	0.40***	0.17
Inherent value demonstration	0.24**	−0.36***	−0.30***	−0.01
Perspective taking	0.36***	0.04	0.009	0.24*
Recurrence	0.06	−0.03	0.17*	−0.16
Warnings * Inherent value demonstration	0.03	−0.17*	−0.28***	0.13
Warnings * perspective taking	−0.18	−0.006	0.08	0.04
Parent’s education	−0.22*	0.24**	0.06	−0.09

The figures in the table are standardized Beta coefficients. Each regression analysis examined the effects of warnings, inherent value demonstration, and their interaction, as well as the main effects of perspective taking and its interaction with inherent value demonstration, recurrence, and parents’ education. Full information on each of the four regression analyses summarized here appears in Tables 2–6 in the supplementary material

*p < 0.05, **p < 0.01, ***p < 0.001

Replicating the findings of the moderated mediation analysis and consistent with the proposed conceptual model, inherent value demonstration had significant interactions with warnings in predicting need thwarting and defiance. As the interaction between inherent value demonstration and warnings as a predictor of need thwarting and of defiance was already examined in the moderated mediation analysis, we do not analyze these interactions here. However, a detailed investigation of these interactions is presented in the supplementary materials (see report of the investigation of the interactions between warning and inherent value demonstration on need thwarting and defiance, followed by Figs. 3 and 4 in the supplementary materials). Results fully replicated the patterns reported in the moderated mediation analysis.

To explore the possibility that problem behavior recurrence moderated the links between warnings and youth experiences and responses, we conducted regression analyses examining the interactions between recurrence and inherent value demonstration. These analyses also controlled for the effects of warnings, inherent value demonstration, perspective taking, the interaction between warnings and inherent value demonstration, parental education, and of course the main effect of recurrence. Results showed no significant interaction effects. Thus, the negative effects of perceived parents’ warnings reactions were observed irrespective of the extent to which the problem behavior occurred before. The results of these regression analyses are presented in Table 7 in the supplementary materials.

### Additional Analyses

We conducted further regression analyses to examine whether the effects of warning are robust and hold when controlling for the main and interactive effects of problem behavior characteristic, as well as demographic and method variables that may reduce or cancel the effects of warnings. These regression analyses controlled for the main and interactive effects of six potential moderating variables: severity of problem behavior participants referred to, number of behaviors engaged in during the last month, the parental figure the child referred to in the questionnaire, parents’ education, adolescent age, and adolescent gender. In addition, each regression included the main effects of warnings, perspective taking, inherent value demonstration, recurrence, and the interaction of warnings with inherent value demonstration. These analyses are presented in Tables 8–13 in the supplemental materials. Results showed that the six potential moderators did not have significant main or interaction effects with warnings. These analyses further support the robustness of our findings across different problem behavior attributes and demographic characteristics.

## Discussion

During adolescence, and particularly in middle adolescence, many adolescents may engage in some problem behaviors (Duell et al., 2018). When parents first find out about this, they may be concerned that the problem behavior may increase over time, perhaps leading to serious deviance and maladjustment. As a result, parents may take preventive actions, such as warning their teen that any further involvement in problem behavior will lead to increased restraints, surveillance, or resource withdrawal. Presently, however, there is very little research on how adolescents experience such warnings and respond to them.

The results of the present study suggest that, as expected, youth experiences and responses to parents’ warnings following the onset of problem behavior depend on the extent to which adolescents perceive their parents as demonstrating the inherit merit of their values in their ongoing behavior (inherent value demonstration). Thus, following parents’ warnings, adolescents experienced their parents’ reactions as more need frustrating and responded with more defiance when parents were low on inherent value demonstration and thus were perceived as failing to authentically realize their values in action.

Many parents and educators may perceive warnings that they will increase restraints and surveillance or withdraw resources following the onset of a problem behavior as a reasonable and benign way to further deter youth engagement in problem behavior. These parental cautions apply to problem behaviors that may hurt their adolescent or others, and therefore can be viewed as attempts to clarify expectations or re-connect adolescents with important values they have evaded. Yet, when parents were perceived as not acting authentically and on the basis of their values, warnings were experienced by many participants in our study as need-thwarting and evoked defiance. These findings suggest that the same parental behavior can be experienced as more or less controlling depending on adolescents’ perceptions of the more general attributes of their parents, such as the extent to which parents consistently enact their values in ways that show that these values are a major source of vitality and meaning in their life.

Notably, the notion that the experience of warnings is a multi-determined subjective phenomenon is consistent with self-determination theory’s view that the extent to which a behavior is perceived as controlling can vary as a function of the person or the situation (Deci & Ryan, 1985). Future research should determine the extent to which adolescents’ experience of controlling behaviors other than warnings also depends on inherent value demonstration or on other general parental attributes.

As expected, inherent value demonstration was also associated positively with greater need support and negatively with more need thwarting and defiance. Importantly, the results demonstrate that inherent value demonstration (alone and as a moderator) had positive correlates even when controlling for the main and moderating effects of the parental reaction of perspective taking, as well as of problem behavior severity, recurrence, and scope, adolescent’s gender and age, and parents’ education. These results suggest that, at least for the population sampled, the positive correlates found for inherent value demonstration are likely to be fairly robust and generalizable.

Along with previous research (Assor et al., 2023), the findings of this study suggest that inherent value demonstration is an important aspect of autonomy-supportive parenting that enables youth to realize (through their parents’ example) the worthiness of parents’ values, and therefore willingly internalize them. Consistent with this view, recent research found that inherent value demonstration was a unique predictor of autonomously internalized values, well-being and resilience. These effects were found even when controlling for the effects of other well-known autonomy supportive practices, such as perspective taking and choice-provision (e.g., Brambila et al., 2015; Cohen et al., 2024; Yu et al., 2023).

Most current conceptualizations and research on autonomy supportive parenting focus on parents’ sensitive response to children’s concerns and preferences, which in turn enhances youth’s autonomous engagement in growth-promoting activities. The concept of inherent value demonstration suggests that such sensitive responses, although of fundamental importance, do not exhaust the domain of autonomy-supportive parenting. Accordingly, to provide optimal support for children’s autonomous functioning and growth, it is important that parents also form autonomously held values (Assor et al., 2023), which they convincingly demonstrate in their ongoing behavior. Said differently, autonomy support is not only about how parents respond to their children, but also about how they conduct themselves in situations that do not involve direct interactions with their children.

The predictions made here regarding the expected positive effects of inherent value demonstration were based on several assumptions about the processes by which inherent value demonstration serves as a protective factor. It was assumed that when parents consistently demonstrate their stated values in their behavior, youth are more likely to respect them and perceive their communications as sincere and worthy. As a result, they tend to perceive parents’ reactions following the onset of problem behaviors as need supporting acts aimed at protecting them. In contrast, when adolescents perceive their parents as lacking a clear and authentic value orientation that they realize in their behavior, youth may be less likely to view their parents’ warning reactions as reflecting deeply held parental values. Instead, they may attribute the warning reactions to parents’ anger or disbelief in the adolescent’s capacity or intention to stay away from trouble. As a result, the warnings of low inherent value demonstration parents are more likely to be perceived as need-thwarting reactions, which in turn may evoke defiance.

In addition, inherent value demonstration may be likely to serve as a particularly strong protective factor against youth anti-social behavior, when parents have strong prosocial values that lie at the core of their sense of authentic inner compass (Assor, 2017; Kaplan & Assor, 2018). In these instances, youth may be more likely to identify with these values, and as a result, avoid anti-social behaviors (Assor, 2011; Assor et al., 2020a). Future research should examine these processes directly.

While parental inherent value demonstration was positively correlated with a number of study variables (i.e., perceiving parents’ reactions as need supporting and not need thwarting, and lack of defiance), it was not associated with adolescents’ reports of ceasing their problem behavior. Thus, while perceived parents’ inherent value demonstration may be associated with youth’s positive interpretation of parents’ behavior, this did not appear to be enough to motivate youth to stop their problem behavior. This may require adolescents to pause and reflect on their motives for involvement in this behavior, its consequences, and what they can do to resist pressures and temptations pushing them to engage in problem behavior. Thus, parents’ inherent value demonstration may not be enough to trigger such a reflective process.

One parental reaction that may promote such a reflective process is parental perspective taking. Indeed, parents’ perceived perspective-taking was the only parenting variable associated with ceasing problem behavior, perhaps because such reactions motivate youth to stop and reflect on their reasons for engaging in the problem behavior. Indeed, the perspective-taking scale includes items that directly refer to parents’ attempts to understand why their child engaged in the problem behavior. In addition, the items also capture youth’s perceptions that their parents accept and care about the feelings underlying their engagement in problem behavior. It is possible that perceiving their parents as acting non-coercively and empathically in trying to understand their motives for engaging in problem behavior may lead adolescents to reflect on why they engage in the problem behavior and the consequences of this behavior. As a result, they may decide to discontinue engaging in it.

Furthermore, adolescents who experience their parents as accepting the feelings underlying their engagement in problem behavior may feel more secure, and therefore allow themselves to engage in honest and non-defensive reflection on their problem behavior. Future research should examine the possibility that perspective taking reactions indeed evoke reflective processes leading to ceasing problem behavior. It would also be worthwhile for research to examine parents’ use of inductive reasoning when youth do not find a clear rationale for avoiding problem behaviors on their own.

Our study used a novel method that allowed us to assess how youth experienced and responded to their parents’ reactions to the most serious problem behavior parents discovered in the recent past (one to four weeks). Focusing on perceived parents’ reactions to a specific problem behavior may increase the likelihood that youth reports get closer to youth’s actual experience and response to parents’ reactions, rather than tapping a general perception of how parents react and how youth feel and respond to their parents. Importantly, identifying youth experiences and responses to parents’ reactions to a *specific* problem behavior may enable us to study potential *cascading processes*, in which involvement in a specific behavior may elicit parents’ need-thwarting reactions to this behavior and thus potentially lead to increased involvement in additional or more severe problem behaviors. As the present research was cross-sectional, it was not possible to examine the potential benefit of our novel method for identifying cascade processes, but future research should examine these processes in a longitudinal design.

Unexpectedly, adolescents with more educated parents experienced less need support and more need thwarting in response to their parents’ reactions to learning about the teen’s problem behavior. It is possible that adolescents with more educated parents are more sensitive to their parents’ responses (Romm et al., 2020). It is also possible that the reactions of more educated parents to the onset of problem behavior may differ in some ways from those of less educated parents. This needs to be examined in future research.

In our analyses, the effect of recurrence of problem behavior was controlled, given its likely association with defiance and need thwarting experiences. Yet, surprisingly, recurrence was associated only with defiance and not with experiencing one’s parents as need frustrating. This may be because many adolescents who reported defiance in response to their parents’ reactions did not experience these reactions as need-thwarting because they already felt detached from their parents and did not expect them to be sensitive to their needs. Consistent with this view, it was found that recurrent problem behavior was positively associated with the number of problem behaviors youth engaged in. It also tended to be negatively related to ceasing problem behavior. Future research should examine whether recurrent problem behavior is associated with a pattern whereby youth stop seeking basic need support from their parents and instead seek such support from deviant peers.

While this study is novel and has many strengths, it also has several limitations that should be noted. First, the correlational design precludes causal interpretations. Participants described their experiences of and responses to parental reactions and, in this sense, the study examined youth’s subjective experience of the causal effects of parents’ reactions. However, it is not possible to know whether youth’s experiences and responses following parents’ reactions do not simply reflect their experiences and responses to their parents before the onset of the problem behavior. Future studies should assess such changes.

Second, the study variables were measured using only youth reports. Adolescents’ experiences of need thwarting, support, and perspective-taking are indeed subjective experiences that are best assessed via adolescents’ reports. However, defiance, and perhaps also ceasing problem behavior, can be usefully assessed with additional informants’ reports. While youth perceptions of parents’ behavior are important, future research could provide a more comprehensive picture of the parenting variables studied here using parent reports and possibly, observation-based measures.

Third, most parental reports of the onset of problem behavior (at least those that they knew of) referred to behaviors occurring in the last week or two. However, some reports referred to behaviors exhibited three to four weeks earlier. This time lag may detract from the accuracy of the reports regarding how parents reacted and adolescents responded to those reactions. Ideally, future research would assess these reactions and responses several hours after the problem behavior is discovered by the parent, and then examine how parents’ and children’s experiences and responses to each other unfold over time. Daily diary studies might be a useful approach to capture these events in real time. Future research might also attempt to identify the time points following the onset of problem behavior in which the assessment of parents’ and children’s reactions best predicts the course of youth involvement in problem behaviors.

Fourth, the present research focused mostly on Israeli Jewish adolescents whose parents completed high school or college. Therefore, the results cannot be generalized to populations of adolescents with less educated parents or to adolescents with other cultural backgrounds. It is also worth noting that no gender differences were found in the processes studied here. Future studies should examine whether findings differ in samples of adolescents varying in age and gender, from different educational and economic backgrounds, and from different cultures. Future studies may also use samples representing the general population.

Fifth, the present study did not include extreme dangerous and illegal acts (e.g., carrying or using a gun, participating in a robbery, drug trafficking, or torturing animals). It is possible that youth may experience and respond to the parental reactions studied differently when these reactions follow extreme delinquent acts. This should be investigated in future research. Also, the assessment of problem behavior recurrence was based only on youth reports. Future studies may assess recurrence also based on reports by parents or available records in the case of illegal behaviors.

Seventh, the study did not examine the effects of contradictory parental reactions to the onset of youth problem behavior. Future research may address this issue. Finally, it would be worthwhile in future studies to examine the effects of warnings and inherent value demonstration together with well-known parenting dimensions such as warmth, behavioral control, structure, monitoring and inductive reasoning or rationale provision. This was not done here, given that the models tested were already quite complex, and the number of teens who reported the onset of recent problem behavior which parents discovered led to reductions in our sample size. It would be important to examine whether the parenting effects found are robust when additional parenting variables are examined simultaneously.

## Conclusion

Although involvement in some problem behavior is normative during adolescence, it is important to understand how parents respond to onset of this behavior and how youth experience and respond to it, as this represents a potentially vulnerable point in adolescent development. The present study highlights the potentially positive role that parents’ inherent value demonstration may play in the ways adolescents experience and respond to their parents’ reactions to the onset of problem behavior. Thus, adolescents who perceived their parents as high on inherent value demonstration experienced their parents’ reactions as less need thwarting and more need-supporting and were less defiant. Parents’ warnings following the onset of problem behavior were more likely to be experienced as need-thwarting and evoke defiance when parents were perceived as failing to demonstrate their values in their ongoing behavior. These findings suggest that parents’ inherent value demonstration may function as a protective factor that enables youth to perceive their parents’ reactions more positively. When parents employ warnings in the absence of high inherent value demonstration, this may be experienced as controlling. The study also highlighted the likely positive role of adolescents’ perceived parents’ perspective-taking reactions to the onset of problem behavior in adolescence, suggesting that they may help prevent continued and increased engagement in more serious problem behavior. Further longitudinal research should examine the potential contribution of inherent value demonstration to parents’ and adolescents’ coping with the onset of problem behavior.

## Supplementary information

Supplementary information
