# Supplementary material for: Parents Value Demonstration as a Determinant of Youth Experiences and Responses to Parents’ Warnings Following the Onset of Risk Behavior
Source: J Youth Adolesc. 2025 Jul 16;54(11):2946–61. doi: 10.1007/s10964-025-02196-7 (PMC12532752; doi:10.1007/s10964-025-02196-7)
Supplement: Supplementary file 1 — Supplementary information [file 10964_2025_2196_MOESM1_ESM.docx]

**Parents’ Value Demonstration as a Determinant of Youth Experiences and Responses to Parents’ Warnings Following the Onset of Problem Behavior**

**Supplementary analyses**

**Pilot Study**

The aim of the pilot study was to determine the severity of each of 19 problem behaviors youth may engage in. So that in the main study, a specially designed computer program can select for each participant the most severe problem behavior she or he engaged in.

To accomplish this aim. We first conducted a pilot study assessing adolescents’ perceptions of the relevance and severity of a wide range of problem behaviors. The severity ratings were then used in the main study to select the most serious problem behavior each participant engaged in during the last month.

The pilot study included 40 Jewish adolescents from the southern part of Israel. They came from the same schools and background as the main study participants. In the first phase, items were selected from available surveys of problem behavior (e.g., Elliott et al., 1985; Kakihara et al., 2010) and following consultation with leading experts. Eight participants (grades 7 – 10) were interviewed regarding the clarity and relevance of these items. Following the interviews, we made some minor modifications to the phrasing of some items. Then, 32 adolescents (M age = 14.72 years, SD = 1.06, range = 13-16, 41.7% female) rated the severity of 29 problem behaviors on a 7-point scale. Item examples are: skipping or missing classes without permission, hanging out at night past curfew, behaving rudely toward older people, lying to parents, internet over-use, smoking cigarettes and drinking alcohol and following an extreme diet. For each behavior, participants were asked to indicate how severe or problematic the behavior was. The scale ranged from *not serious* (1) to *extremely serious* (7). The items appear in the supplementary material (Table 1).

Inspection of severity ratings suggested that most items were considered mildly serious, and that there was considerable variability in severity scores. To evaluate the validity of these ratings, we also assessed the severity of the same items with a sample of 30 first year college students and 15 parents of adolescents. Results showed an ICC of 0.95 between the mean severity ratings of the adolescent and parent groups, and an ICC of 0.70 between the mean severity ratings of the adolescent and student groups. These coefficients suggest that adolescent ratings used to determine the severity of the behaviors examined in our study reflect severity perceptions common to different age groups.

To assess whether behavior severity affects the findings pertaining to the main hypotheses, it was necessary to create an index of the severity of the individualized problem behavior each participant referred to. To creates this index, we averaged the severity scores of all items to create a *severity* scale. Cronbach alpha was .87 (*M* = 4.91, *SD* = .97), indicating that the items reflect one dimension.

**Table 1**

*Frequencies of the problem behaviors presented to participants.*

| **Item #** | **Item content** | **Frequency** |
| --- | --- | --- |
| 1 | I behaved rudely towards or made fun (in a demeaning or cruel way) of older people (e.g. relatives, teachers, sellers, etc.). | 22.9 |
| 2 | I was so involved with on-line social media that I completely neglected important activities and did not respond to my parents. | 17.1 |
| 3 | I skipped or missed classes (without my parents’ and/or school staff permission). | 8.6 |
| 4 | I hung outside at night way past curfew hour – against my parents’ will. | 7.6 |
| 5 | I hitchhiked with strange people | 7.2 |
| 6 | I dieted excessively or binged and purged. | 4.8 |
| 7 | I hit, bullied, threatened, or humiliated another kid. | 2.9 |
| 8 | I consumed marijuana, cannabis, hashish or grass. | 2.9 |
| 9 | I smoked cigarettes, electronic cigarettes, or hookah. | 2.9 |
| 10 | I dressed up in a way my parents’ think is too revealing and exaggerated. | 2.9 |
| 11 | I took money or other things without permission from places that are not my house. | 1.9 |
| 12 | I hung out with peers who spread very embarrassing things about another child. | 1.9 |
| 13 | I destroyed, ruined, or damaged property that doesn't belong to me (Such as: windows, streetlights, benches, cars, gardens, etc.). | 1.9 |
| 14 | I took money or other things without permission from my home or family members. | 1.9 |
| 15 | I hung out with kids who destroyed, ruined, or damaged property that doesn't belong to me (Such as: windows, streetlights, benches, cars, gardens, etc.). | 1.9 |
| 16 | I hung out with peers who hit, bullied, threatened, or humiliated another kid. | 1.9 |
| 17 | I was in touch with older people I don't know through the internet, WhatsApp, snapchat etc. | 1.9 |
| 18 | I drank alcohol excessively. | 1.9 |
| 19 | I did sexual things that adults around me view as unacceptable. | 1 |
| 20 | I lied to my parents about something important, such as where I'm going or who I'm hanging out with. | 1 |
| 21 | I hung out with kids that did sexual things that hurt other kids or said things that made them feel very uncomfortable. | 1 |
| 22 | I hung out with peers who spread embarrassing photos of someone on the internet or on an app (with sexual or other contents) - without their permission. | 1 |
| 23 | I was part of a group that excluded another kid. | 1 |
| 24 | I spread embarrassing photos of someone on the internet or on an app (with sexual or other contents) - without their permission | 0 |
| 25 | I humiliated or spread very embarrassing things about another child | 0 |
| 26 | I did or said sexual things that hurt other kids or made them feel very uncomfortable. | 0 |
| 27 | I played dangerous games or done dangerous things without parental permission or professional guidance. | 0 |
| 28 | I hung out in dangerous places. | 0 |
| 29 | I watched movies that have excessive sexual content | 0 |

**Table 2**

*Results of CFA of the four parental behaviors*

|  |  |  | **β** | ***B*** | ***S.E.*** | ***t*** | ***P*** |
| --- | --- | --- | --- | --- | --- | --- | --- |
| Warnings | 🡪 | 1. My parents said that if I continue to behave this way, they will have to make more stringent rules, and they will be very strict about keeping them | .71 | 1.17 | .17 | 6.78 | <.001 |
| Warnings | 🡪 | 1. My parents said that if I continue to behave this way, they will have to ask me to give them detailed information about what I do, who I hang out with, where I go, and when I return. | .75 | .92 | .11 | 8.09 | <.001 |
| Warnings | 🡪 | 1. My parents told me that if I continue to behave this way, they will have to stop giving me an allowance(money) or buy me things I want or disconnect my cell phone or internet connection | .70 | .85 | .12 | 6.78 | <.001 |
| Warnings | 🡪 | 1. My parents said that if I continue to act this way, they will have to limit the places I can go out to (for entertainment) and the number of times I can go out. | .77 | .90 | .11 | 7.65 | <.001 |
| IVD | 🡪 | parc1_IVD | .78 | .75 | .09 | 8.05 | <.001 |
| IVD | 🡪 | parc2_IVD | .92 | 1.32 | .16 | 8.05 | <.001 |
| IVD | 🡪 | parc3_IVD | .70 | .81 | .11 | 7.16 | <.001 |
| Taking perspective | 🡪 | 1. My parents tried to listen to my side and wanted to hear my opinion | .75 | 1.19 | .21 | 5.44 | <.001 |
| Taking perspective | 🡪 | 1. My parents really cared about how I felt about this issue | .74 | .88 | .13 | 6.47 | <.001 |
| Taking perspective | 🡪 | 1. My parents really tried to understand what I was feeling and what made me act this way | .70 | .91 | .14 | 6.22 | <.001 |
| Taking perspective | 🡪 | 1. My parents showed me that even though I misbehaved, they know and trust that I am a good person and I can improve my ways. | .60 | .84 | .15 | 5.44 | <.001 |

**Table 3**

*Regression analysis: warnings, perspective taking and recurrence as predictors of ceasing the problem behavior*

|  |  | ***B*** | ***S.E*** | ***t*** | ***p*** | ***F*** |
| --- | --- | --- | --- | --- | --- | --- |
| Warnings |  | .15 | .09 | 8.52 | .11 | *F*(7, 91) = 1.96 *p*=.06, *R^2^*=.13 |
| IVD |  | -.008 | .13 | -.05 | .95 |  |
| Perspective Taking |  | .18 | .08 | 2.23 | .02 |  |
| Recurrence |  | -.25 | .16 | -1.52 | .13 |  |
| Warnings * IVD |  | .15 | .12 | 1.14 | .25 |  |
| Warnings * Perspective taking |  | .02 | .07 | .38 | .70 |  |
| Parental education |  | -.05 | .06 | -.85 | .39 |  |

**Table 4**

*Regression analysis: warnings, perspective taking and recurrence as predictors of defiance*

|  |  | ***B*** | ***S.E*** | ***t*** | ***p*** | ***F*** |
| --- | --- | --- | --- | --- | --- | --- |
| Warnings |  | .38 | .08 | 4.66 | <.001 | *F*(7, 91) = 8.61, *p*<.001, *R^2^*=.40 |
| IVD |  | -.44 | .12 | -3.53 | <.001 |  |
| Perspective Taking |  | -.005 | .07 | -.07 | .94 |  |
| Recurrence |  | .34 | .15 | 2.30 | .02 |  |
| Warnings * IVD |  | -.40 | .11 | -3.44 | <.001 |  |
| Warnings * Perspective taking |  | .06 | .07 | .96 | .33 |  |
| Parental education |  | .04 | .06 | .80 | .42 |  |

**Table 5**

*Regression analysis: warnings, perspective taking and recurrence as predictors of need support*

|  |  | ***B*** | ***S.E*** | ***t*** | ***p*** | ***F*** |
| --- | --- | --- | --- | --- | --- | --- |
| Warnings |  | -.009 | .08 | -.01 | .99 | *F*(7, 91) = 6.37, *p*<.001, *R^2^*=.33 |
| IVD |  | .36 | .12 | 2.81 | .005 |  |
| Perspective Taking |  | .28 | .07 | 3.75 | <.001 |  |
| Recurrence |  | .04 | .15 | .28 | .77 |  |
| Warnings * IVD |  | .10 | .11 | .85 | .39 |  |
| Warnings * Perspective taking |  | -.12 | .06 | -1.84 | .11 |  |
| Parental education |  | -.17 | .06 | -2.69 | .008 |  |

**Table 6**

*Regression analysis: warnings, perspective taking and recurrence as predictors of need thwarting*

|  |  | ***B*** | ***S.E*** | ***t*** | ***p*** | ***F*** |
| --- | --- | --- | --- | --- | --- | --- |
| Warnings |  | .32 | .05 | 5.87 | <.001 | *F*(7, 91) = 12.29, *p*<.001, *R^2^*=.49 |
| IVD |  | -.38 | .08 | -4.51 | <.001 |  |
| Perspective Taking |  | .02 | .04 | .44 | .65 |  |
| Recurrence |  | -.03 | .09 | -.37 | .70 |  |
| Warnings * IVD |  | -.16 | .08 | -2.07 | .04 |  |
| Warnings * Perspective taking |  | -.003 | .04 | -.07 | .93 |  |
| Parental education |  | .12 | .04 | 3.08 | .002 |  |

**Table 7**

*Regression analyses predicting adolescents’ experiences of need support and need thwarting, ceasing the problem behavior, and defiance*

| Variables in regression | Need support | Need thwarting | Ceasing the risk behavior | Defiance |
| --- | --- | --- | --- | --- |
| Warnings | .02 | .52*** | .17 | .42*** |
| IVD | .24** | -.36*** | -.002 | -.30*** |
| Perspective taking | .32*** | .10 | .24* | .03 |
| Recurrence | .05 | -.01 | -.16 | .18* |
| Warnings * IVD | .02 | -.16* | .13 | -.27** |
| Warnings * recurrence | -.13 | .15 | .005 | .14 |
| Parents' education | -.23* | .25*** | -.09 | .06 |

**Table 8**

*Regression analyses predicting adolescents’ experiences of need support and need thwarting, ceasing the problem behavior, and defiance*

| Variables in regression | Need support | Need thwarting | Ceasing the risk behavior | Defiance |
| --- | --- | --- | --- | --- |
| Warnings | .02 | .46*** | .20 | .36*** |
| IVD | .24** | -.36*** | .003 | -.30*** |
| Perspective taking | .36*** | .04 | .23* | .004 |
| Recurrence | .06 | -.02 | -.17 | .18* |
| Warnings * IVD | .03 | -.17* | .13 | -.28*** |
| Parental education | -.22* | .24* | -.09 | .06 |
| Warnings * Parental education | .07 | .10 | -.15 | .18 |

**Table 9**

*Regression analyses predicting adolescents’ experiences of need support and need thwarting, ceasing the problem behavior, and defiance*

| Variables in regression | Need support | Need thwarting | Ceasing the risk behavior | Defiance |
| --- | --- | --- | --- | --- |
| Warnings | .03 | .48*** | .16 | .40*** |
| IVD | .26** | -.38*** | .004 | -.30*** |
| Perspective taking | .36*** | .04 | .26* | -.004 |
| Recurrence | .08 | -.06 | -.14 | .18* |
| Warnings * IVD | .05 | -.20* | .14 | -.28*** |
| Child's gender | -.04 | .07 | .10 | .06 |
| Warnings * Child's gender | -.11 | .17 | -.09 | -.01 |

**Table 10**

*Regression analyses predicting adolescents’ experiences of need support and need thwarting, ceasing the problem behavior, and defiance*

| Variables in regression | Need support | Need thwarting | Ceasing the risk behavior | Defiance |
| --- | --- | --- | --- | --- |
| Warnings | .002 | .52*** | .15 | .41*** |
| IVD | .24* | -.36*** | .002 | -.28*** |
| Perspective taking | .48*** | -.02 | .24* | -.13 |
| Recurrence | .07 | -.03 | -.16 | .18* |
| Warnings * IVD | .02 | -.17* | .14 | -.26*** |
| Parental figure | -.19 | .04 | .02 | .18 |
| Warnings * Parental figure | -.05 | .15 | .004 | .16 |

**Table 11**

*Regression analyses predicting adolescents’ experiences of need support and need thwarting, ceasing the risk behavior, and defiance*

| Variables in regression | Need support | Need thwarting | Ceasing the risk behavior | Defiance |
| --- | --- | --- | --- | --- |
| Warnings | .002 | .52*** | .15 | .41*** |
| IVD | .24* | -.36*** | .002 | -.28*** |
| Perspective taking | .48*** | -.02 | .24* | -.13 |
| Recurrence | .07 | -.03 | -.16 | .18* |
| Warnings * IVD | .02 | -.17* | .14 | -.26*** |
| Severity of problem behavior | -.19 | .04 | .02 | .18 |
| Warnings * Severity | -.05 | .15 | .004 | .16 |

**Table 12**

*Regression analyses predicting adolescents’ experiences of need support and need thwarting, ceasing the problem behavior, and defiance*

| Variables in regression | Need support | Need thwarting | Ceasing the risk behavior | Defiance |
| --- | --- | --- | --- | --- |
| Warnings | .03 | .48*** | .17 | .36*** |
| IVD | .18 | -.28*** | .009 | -.20* |
| Perspective taking | .37*** | -.03 | .24* | -.007 |
| Recurrence | .09 | -.07 | -.14 | .13 |
| Warnings * IVD | .05 | -.17* | .10 | -.28*** |
| Number of behavior problems | -.19 | .24** | -.03 | .28*** |
| Warnings * Number of behavior  problems | -.02 | .07 | -.13 | .08 |

**Table 13**

*Regression analyses predicting adolescents’ experiences of need support and need thwarting, ceasing the problem behavior, and defiance*

| Variables in regression | Need support | Need thwarting | Ceasing the risk behavior | Defiance |
| --- | --- | --- | --- | --- |
| Warnings | .03 | .51*** | .20 | .38*** |
| IVD | .25** | -.37*** | .001 | -.30*** |
| Perspective taking | .38*** | .01 | .23* | -.005 |
| Recurrence | .06 | -.03 | -.11 | .14 |
| Warnings * IVD | .03 | -.17* | .12 | -.28*** |
| Child's age | .06 | -.04 | -.18 | .12 |
| Warnings * Child's age | -.02 | .11 | -.13 | .03 |

Investigation of the effects of the interactions between warnings and IVD reported in Table 3

Table 3 shows that, consistent with the proposed conceptual model, IVD had significant interactions with warnings in predicting need thwarting and defiance. These findings indicate that, as expected, IVD moderated the link between warnings and experiencing parents’ reactions as need thwarting, as well as between warnings and defiant response to parents

To further investigate the interaction between warnings and IVD on need-thwarting and defiance, and following Aiken and West (1991), we estimated three regression lines for perceived warnings on defiance and on need-thwarting at three levels of perceived IVD. Specifically, regression lines were estimated for a relatively high level (+1 SD), moderate (mean), and relatively low level of IVD (-1 SD). Figure 1 presents the interaction between perceived parental warnings and adolescents’ perceptions of parents’ IVD as predictors of adolescents’ experience of parents’ reactions as need thwarting. Tests of simple slopes indicated that, as expected, the association between perceived warnings and need thwarting increased the more youth perceived their parents as low on IVD (For low IVD: β = .66, *t* = 5.64, *p* < .001): for medium IVD : (β = .49, *t* = 6.05, *p* < .001); for high IVD: high (β = .33, *t* =2.94, *p* =.004).

Figure 2 presents the interaction between parental warnings and adolescents’ perceptions of parents’ IVD as predictors of adolescents’ defiant response to parents’ reactions. Tests of simple slopes indicated that perceived warnings were positively and significantly associated with defiance only when perceived IVD was low (β = .68, t = 5.36, *p* < .001) or average (β **=** .40, *t* = 4.56, *p* < .001). The association between warnings and defiance was not significant when IVD was high (β = .12, *t* = 1.01, *p* =.31).

**Figure 3**

*IVD as a moderator of the link between warnings and the experience of parents’ reactions as need thwarting*

Warning to increase restraints, surveillance and withdraw resources

*Note:* The slopes were assessed with the main effects of of warnings, IVD, perspective taking, recurrence, and parents’ education controlled.

**Figure 4**

*IVD as a moderator of the link between warnings and defiant response to parents’ reactions*

Warning to increase restraints, surveillance and withdraw resources

*Note:* The slopes were assessed with the main effects of warnings, IVD, perspective taking, recurrence, and parents’ education controlled
